# Function-Triggering Antibodies to the Adhesion Molecule L1 Enhance Recovery after Injury of the Adult Mouse Femoral Nerve

**DOI:** 10.1371/journal.pone.0112984

**Published:** 2014-11-13

**Authors:** Daria Guseva, Gabriele Loers, Melitta Schachner

**Affiliations:** 1 Zentrum für Molekulare Neurobiologie, Universitätsklinikum Hamburg-Eppendorf, Hamburg, Germany; 2 Cellular Neurophysiology, Hannover Medical School, Hannover, Germany; 3 Center for Neuroscience, Shantou University Medical College, Shantou, China; 4 W. M. Keck Center for Collaborative Neuroscience and Department of Cell Biology and Neuroscience, Rutgers University, Piscataway, New Jersey, United States of America; National Cancer Institute, United States of America

## Abstract

L1 is among the few adhesion molecules that favors repair after trauma in the adult central nervous system of vertebrates by promoting neuritogenesis and neuronal survival, among other beneficial features. In the peripheral nervous system, L1 is up-regulated in Schwann cells and regrowing axons after nerve damage, but the functional consequences of this expression remain unclear. Our previous study of L1-deficient mice in a femoral nerve injury model showed an unexpected improved functional recovery, attenuated motoneuronal cell death, and enhanced Schwann cell proliferation, being attributed to the persistent synthesis of neurotrophic factors. On the other hand, transgenic mice over-expressing L1 in neurons led to improved remyelination, but not improved functional recovery. The present study was undertaken to investigate whether the monoclonal L1 antibody 557 that triggers beneficial L1 functions *in vitro* would trigger these also in femoral nerve repair. We analyzed femoral nerve regeneration in C57BL/6J mice that received this antibody in a hydrogel filled conduit connecting the cut and sutured nerve before its bifurcation, leading to short-term release of antibody by diffusion. Video-based quantitative analysis of motor functions showed improved recovery when compared to mice treated with conduits containing PBS in the hydrogel scaffold, as a vehicle control. This improved recovery was associated with attenuated motoneuron loss, remyelination and improved precision of preferential motor reinnervation. We suggest that function-triggering L1 antibodies applied to the lesion site at the time of injury over a limited time period will not only be beneficial in peripheral, but also central nervous system regeneration.

## Introduction

The neural cell adhesion molecule L1 is a glycoprotein of the immunoglobulin superfamily expressed in most, if not all, neurons in the central and peripheral nervous systems of mammals. During development, L1 is targeted to the surface of developing neurites and growth cones and mediates axonal outgrowth, fasciculation and guidance as well as neuronal migration and survival [1]–[5]. L1 is expressed by glial cells in the peripheral, but not central nervous system. Mutations in the L1 gene lead to abnormal nervous system development and dysfunctions in mammals, insects and worms. L1 is also implicated in nervous system regeneration after injury in adult vertebrates. After spinal cord injury in zebrafish, the expression of L1.1, a homolog of the mammalian L1, is increased in successfully regenerating descending axons but not in ascending projections that fail to regenerate, and suppression of L1.1 by anti-sense morpholino application in the injured spinal cord reduces the spontaneous locomotor recovery [6]. Similar to the poorly regenerating neurons in zebrafish, mammalian neurons fail to up-regulate L1 expression after trauma [7], [8]. When L1 in central nervous system neurons and glia is ectopically expressed via viral transduction [9] or when the regeneration-adverse environment is overcome by application of exogenous L1 [10], recovery from spinal cord injury is enhanced. Furthermore, L1 overexpressing neural stem cells as well as adeno-associated virus encoding the neuronal isoform of full-length L1 ameliorate the functional deficits in animal models of Parkinson's, Huntington's and Alzheimer's diseases [11]–[15]. These results indicate that L1 is beneficial for recovery after acute trauma and during chronic degenerative processes.

In the peripheral nervous system, L1 is expressed in axons and Schwann cells during embryonic and early postnatal development, and remains expressed by non-myelinating Schwann cells in the adult [16]–[19]. L1 mediates the contact between axons and Schwann cells at early stages of myelination *in vitro* [20]–[22]. Peripheral nerve injury in adult rodents leads to up-regulation of L1 expression in myelin-competent Schwann cells distal to the injury site and in regenerating neuronal cell bodies and their axons [18], [23], [24]. In L1-deficient mice, non-myelinating Schwann cells generate abnormal processes and fail to appropriately ensheath small caliber axons [25], [26]. Unexpectedly, these mice exhibit better functional recovery after femoral nerve injury paralleled by enhanced Schwann cell proliferation, but reduced propensity for remyelination [27], whereas transgenic overexpression of L1 in neurons under control of the Thy-1 promoter enhanced myelination, but did not improve motor recovery after femoral nerve injury [28]. *In vitro*, application of recombinant L1 to Schwann cells increased their motility, and, *in vivo*, virally L1-transduced Schwann cells myelinated regrown/sprouted axons after spinal cord injury, while retaining their motility [29]. Based on these observations we asked whether an L1 function-triggering monoclonal antibody reacting with an epitope in the third fibronectin type III homologous repeat in the extracellular domain of L1 would improve motor recovery after femoral nerve transection and repair in adult mice [30]–[33]. In the present study, we found not only better functional recovery in mice treated with this antibody, but also attenuated motoneuron loss, enhanced myelination of regrown/sprouted axons, and improved precision of preferential motor reinnervation.

## Materials and Methods

### L1 monoclonal antibody 557

L1 monoclonal antibody 557 (L1 Ab 557) was prepared against L1 from adult mouse brain in Lou×Sprague-Dawley F1 hybrid rats as described [34]. The specificity of antibody 557 for the cell adhesion molecule L1 has been documented [35], [36]. The function-triggering, i.e. agonistic action of the Fab fragment of antibody 557 has also been reported [37].

Application of peptides and proteins via filling of a conduit is sufficient to deliver a protein or peptide of interest to the injury site and has been shown in several studies performed in our group [33], [38], [39]. Based on previous studies we expect that the half-life of the 557 antibody is similar to the half-life of other antibodies, such as the anti-nerve growth factor receptor antibody which has a plasma half-life of 5–6 days [40]. Full antibodies were applied in our study with the reasonable assumption that after a one-time application within the hydrogel cuff the full antibodies would be present over a longer time period than the Fab fragments, since the larger full-sized antibodies penetrate into the tissue more slowly than the Fab fragments and are also cleared more slowly. In view of translational aims this would not prohibit the use of these antibodies, since there a robust functional impact on regeneration seen with the IgGs, an effect that cannot be attributed to non-specific functions. Furthermore, we decided to take advantage of the ‘clustering’ capability of IgGs that is known to enhance the functional impact on cell surface receptors, an effect that is achieved by IgG's and not by Fabs.

### Ethics Statement

All experiments were conducted in accordance with the German and European Community laws on protection of experimental animals, and all procedures used were approved by the responsible committee of The State of Hamburg (TvG 05/65). Experiments were carried out and the manuscript prepared following the ARRIVE guidelines for animal research.

### Animals and surgical procedures

Before and after the experiments, three-month-old C57BL/6J mice were kept under standard laboratory conditions, with food and tap water *ad libitum*, and an artificial 12 h light/dark cycle.

Surgery was performed as described [33]. The mice were anesthetized by intraperitoneal injections of 0.4 mg/kg fentanyl (Fentanyl-Janssen, Janssen, Neuss, Germany), 10 mg/kg droperidol (dehydrobenzperidol, Janssen) and 5 mg/kg diazepam (Valium 10 Roche, Hoffman – La Roche, Grenzach-Wyhlen, Germany). The right femoral nerve was exposed and transected approximately 3 mm proximal to the bifurcation of the sensory (saphenous) and motor (quadriceps, which is a mixed motor and sensory nerve branch) branches. The cut ends of the nerve were inserted into a polyethylene tube (3 mm length, 0.58 mm inner diameter, Becton Dickinson, Heidelberg, Germany) and fixed with a single epineural 11-0 nylon stitch (Ethicon, Norderstedt, Germany) so that a 2 mm gap was present between the proximal and distal nerve stumps. The tube was filled with L1 Ab 557 diluted 1∶1 with scaffold peptide (BD PuraMatrix Peptide Hydrogel, BD Bioscience) in phosphate buffered saline pH 7.4 (PBS) to give a final concentration of 400 µg/ml when applied to the tube conduit. PBS was used as vehicle control. Diffusion of a protein containing the extracellular domain of the cell adhesion molecule L1 coupled to the Fc part of human IgG which is similar in size to a full length IgG was shown to penetrate/diffuse several mm in the lesioned spinal cord [10] and we assume a similar extent of diffusion here. Experiments with peptides mimicking the glycans polysialic acid and HNK-1 [33], [38], [39] have shown that the used application method is suitable and sufficient to deliver proteins and peptides to the injured nerve. After surgery, the skin was closed with 6-0 sutures (Ethicon). To prevent hypothermia, the mice were then kept in a warm room (35°C) until full recovery from anesthesia.

### Analysis of motor function

**Figure 1:**
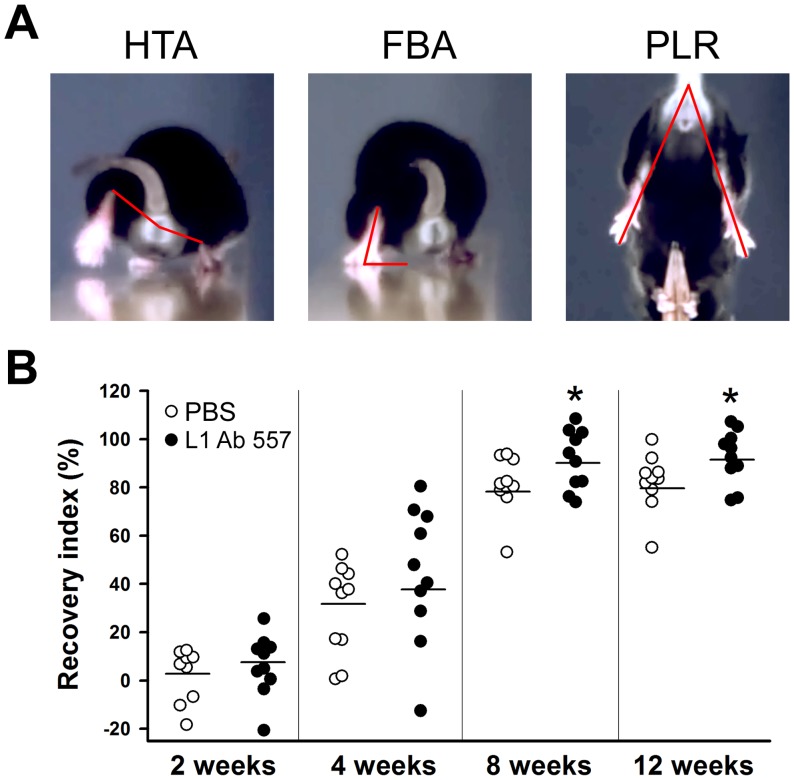
Time-course of motor recovery after femoral nerve injury. (**A**) Representative images of measured parameters: heels-tail angle (HTA), foot-base angle (FBA) and protraction length ratio (PLR). (**B**) Analysis of the recovery indices at 2, 4, 8 and 12 weeks after femoral nerve transection. 10 mice per group were analyzed. Asterisks indicate differences between PBS treated and L1 Ab 557 treated groups at 8 and 12 weeks after injury (*p<0.05, one-way ANOVA with Tukey's *post hoc* test).

Analysis was performed over a time-period of 12 weeks using a quantitative video-based approach (single-frame motion analysis) [32]. To evaluate locomotor ability during ground locomotion, mice were trained to perform a beam-walking test. In this test, the animal walks unforced from one end of a horizontal beam (length 1000 mm, width 40 mm) towards its home cage located at the other end of the beam. For all mice, a rear view of one walking trial was captured prior to the operation with a high-speed camera (A602fc, Basler, Ahrensburg, Germany) at 100 frames per second and stored on a personal computer in Audio Video Interleaved (AVI) format. The recordings were repeated 1, 2, 4, 8 and 12 weeks after nerve transection. The video sequences were examined using SIMI-Motion 7.0 software (SIMI Reality Motion Systems, Unterschleissheim, Germany). Selected frames in which the animals were seen in defined phases of the step cycle were used for measurements of two parameters: the heels-tail angle and the foot-base angle (HTA and FBA, respectively, Fig. 1A) as described [32]. Both parameters are directly related to the ability of the quadriceps muscle to keep the knee joint extended during contralateral swing phases. As a relative measure of functional recovery at different time-points after nerve injury, we calculated the stance recovery index, which is a mean of the recovery index (RI) for the HTA and the FBA. The index for each angle is calculated in percent as:
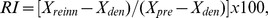
where X _pre_, X _den_ and X _reinn_ are values prior to surgery, during the state of denervation (7 days after injury), and at any given time-point during reinnervation, respectively.

A third parameter, the limb protraction length ratio (PLR, Fig. 1A), was evaluated from video-recordings of voluntary pursuit movements of the mice [32]. The mouse, when held by its tail and allowed to grasp a pencil with its forepaws, tries to catch the object with its hindpaws and extends simultaneously both hindlimbs. In non-injured animals, the relative length of the two extremities, as estimated by lines connecting the most distal mid-point of the extremity with the anus, is approximately equal and the PLR (ratio of the right to left limb length) is close to 1. After denervation, the limb cannot extend maximally, and the PLR increases significantly above 1.

### Retrograde labeling of motoneurons

Twelve weeks after nerve transection the animals were anaesthetized with fentanyl, droperidol and diazepam for retrograde labeling of regenerated motoneurons [33]. After exposure of the right femoral nerve, a piece of Parafilm (Pechiney Plastic Packaging, Chicago, IL, USA) was inserted underneath the nerve and the two nerve branches were transected approximately 5 mm distal to the bifurcation. Fluorescent retrograde tracers were applied to the cut nerve ends in powder form: Fluoro-ruby (tetramethylrhodamine dextran, Molecular Probes, Leiden, The Netherlands) to the sensory branch and Fast Blue (EMS-Chemie, Großumstadt, Germany) to the motor branch. Thirty minutes after dye application, the nerve stumps were rinsed with PBS and the wound was closed. The same labeling procedure was applied to mice which had not been subjected to injury.

### Preparation of tissue for morphological analyses

Seven days after retrograde labeling the mice were anesthetized by intraperitoneal injection of 16% sodium pentobarbital solution (Narcoren, Merial, Hallbergmoos, Germany, 5 µl/g body weight) and transcardially perfused with 4% formaldehyde in 0.1 M sodium cacodylate buffer, pH 7.3. The lumbar spinal cords were removed, post-fixed overnight at 4°C in the perfusion solution and then immersed in 15% sucrose solution in 0.1 M cacodylate buffer, pH 7.3, for one day at 4°C. Thereafter the tissue was frozen for 2 min in 2-methyl-butane (isopentane, Carl Roth, Karlsruhe, Germany) pre-cooled to −30°C. For sectioning, spinal cord segments were attached to a cryostat specimen holder using TissueTek (Sakura Finetek Europe, Zoeterwoude, The Netherlands). Serial cross-sections of 25 µm thickness were cut on a cryostat (Leica CM3050, Leica Instruments, Nußloch, Germany) and attached to SuperFrostPlus glass slides (Carl Roth). The sections were air-dried for at least 1 h at room temperature and mounted in anti-fading medium (Fluoromount G, Southern Biotechnology Associates, Biozol, Eching, Germany).

For the analysis of re-myelination, femoral nerves were dissected from the animals fixed by perfusion and post-fixed in 1% osmium tetroxide (Polysciences Europe, Eppelheim, Germany) in 0.1 M sodium cacodylate buffer, pH 7.3, for 1 h at room temperature, dehydrated and embedded in resin according to standard protocols. One µm-thick cross-sections from the motor and sensory nerve branches were cut at a distance of approximately 3 mm distal to the bifurcation and stained with 1% toluidine blue/1% borax in distilled water for analysis of axons in the regenerated nerve branches.

### Analysis of retrogradely labeled motoneurons

Serial cross-sections of spinal cords were examined under a fluorescence microscope (Axiophot 2, Zeiss, Oberkochen, Germany) with appropriate filter sets. Each section was examined using a 40× objective by focusing through the section thickness starting from the top surface. All profiles, except those visible at the top surfaces of sections, were counted [33]. This stereological principle prevents double counting of labeled cells, thus allowing precise evaluation of cell numbers.

### Immunofluorescence labeling

Immunofluorescence staining was performed as described [28], [41] using antibodies at optimal dilutions (Table 1).

**Table 1:** Primary antibodies used in this study.

Antibody	Cellular phenotypes/structures recognized	Host	Code/clone	Dilution	Source
Choline acetyltransferase (ChAT)	Cholinergic synaptic terminals	Goat	AB144P	1∶100	Chemicon, Hofheim, Germany
Vesicular GABA transporter (VGAT)	Inhibitory (GABA- and glycinergic) synaptic terminals	Mouse	131 011	1∶1,000	Synaptic Systems, Göttingen, Germany

For analyses of perisomatic terminals, spinal cord sections containing retrogradely labeled motoneurons (see above) were freed from the coverslips and mounting medium by soaking in PBS. Antigen retrieval was performed by immersion into 0.01 M sodium citrate solution, pH 9.0, heated to 70°C in a water bath for 30 min. Blocking of non-specific binding sites was then performed using PBS containing 0.2% Triton X-100 (Sigma-Aldrich, St. Louis, MO), 0.02% sodium azide (Merck, Darmstadt, Germany) and 5% normal goat or normal donkey serum (Jackson ImmunoResearch Europe, Suffolk, UK) for 1 h at room temperature. Incubation with primary antibodies against VGAT or ChAT (Table 1), diluted in PBS containing 0.5% lambda-carrageenan (Sigma-Aldrich) and 0.02% sodium azide, was carried out at 4°C for 3 days. For a given antigen, all sections were stained in the same solution kept in screw-capped staining plastic jars (capacity 35 ml, 10 slides; Roth). After washing in PBS, appropriate Cy3-conjugated secondary antibodies (Jackson ImmunoResearch Europe) diluted 1∶200 in PBS-carrageenan solution were applied for 2 h at room temperature. Specificity of staining was controlled by omitting the primary antibody or replacing it by an equivalent amount of non-immune IgG or serum derived from the same species as the specific antibody. These controls did not show labeling.

### Analyses of motoneuron size and numbers of perisomatic synaptic terminals

Sections containing retrogradely labeled motoneurons and additionally stained for ChAT and VGAT were used to estimate soma areas and motoneuron perisomatic coverage [27], [28], [33], [42]. Linear density (number per unit area) of perisomatic terminals was estimated for motoneurons that had correctly projected to the motor nerve branch of the femoral nerve (identified by Fast Blue back-labeling). Stacks of images of 1 µm thickness were obtained on a confocal microscope (Leica DM IRB, Wetzlar, Germany) using a 63× oil immersion objective and digital resolution of 1024×1024 pixels. One image per cell at the level of the largest cell body cross-sectional area was used to measure soma area, and number of perisomatic puncta. These measurements were performed using the image tool software Fiji (http://fiji.sc/Fiji) and the LSM image browser (Zeiss) was used to select the image with the largest cell body cross section.

### Analyses of axon numbers in the regenerated nerve branches

Total numbers of myelinated axons per nerve were estimated in semithin cross-sections on an Axioskop microscope (Zeiss) equipped with a motorized stage and Neurolucida software-controlled computer system (MicroBrightField Europe, Magdeburg, Germany) using a 100× oil objective [33]. Axonal and nerve fiber diameters were measured in a random sample from each section. For sampling, a grid with line spacing of 60 µm was projected into the visual field using the Neurolucida software. Selection of the reference point (zero coordinates) of the grid was random. For all myelinated axons crossed by or touching the vertical grid lines through the sections, mean orthogonal diameters of the axon (inside the myelin sheath) and of the nerve fiber (including the myelin sheath) were measured. The mean orthogonal diameter is calculated as a mean of the line connecting the two most distal points of the profile (longest axis) and the line passing through the middle of the longest axis at right angle [43]. The degree of myelination was estimated by the ratio axon to fiber diameter (g-ratio).

### Statistical analyses

All numerical data are presented as group mean values with SEM. Parametric or nonparametric tests were used for comparisons, as indicated in the text and figure legends. For parametric tests, the mean was used as a representative value if two or more measurements per parameter and animal were performed. Thus, for all these comparisons the degree of freedom was determined by the number of animals. Analyses were performed using the SYSTAT 9 software package (SPSS, Chicago, IL). The threshold value for acceptance of differences was 5%. The size of each sample (analyzed numbers of animals, axons, and neurons) is indicated in the figures or their captions.

## Results

### Enhanced motor recovery by function-triggering monoclonal L1 antibody

Transection of the femoral nerve induces changes in the heels-tail angle (HTA) and foot-base angle (FBA) (Fig. 1A) as well as changes of locomotor functions. In mice treated with PBS (vehicle control) and the function triggering monoclonal L1 antibody 557 (hereafter called L1 Ab 557 [35]), the degree of disability at 1 week after surgery was similar in both experimental groups and thereafter gradually returned to the pre-operative values. As estimated by the stance recovery index (this index allows comparison of values prior to injury, during the state of denervation, and at any given time-point during reinnervation and is a measure of the individual degree of recovery calculated for the HTA and FBA), repair was better in animals treated with L1 Ab 557 (92±10%) compared with PBS treated animals (79±12%, p<0.05, *t* test, Fig. 1B). The combined locomotor estimates reveal a better functional outcome in mice treated with L1 Ab 557 at 8 and 12 weeks after injury.

In addition to ground locomotion, we also evaluated the animals' ability to extend the knee joint during movements without body weight support using the “pencil” test [32]. This ability is determined by the limb protraction length ratio (PLR) which measures the (length of the non-injured hindlimb over the length of injured limb during maximum extension) (Fig. 1A). The degree of disability at 1 week after surgery was again similar in the two experimental groups. While the recovery index of PLR showed no difference between groups also at later time points, the combined results estimated by HTA and FBA showed a better functional outcome in mice treated with L1 Ab 557.

### Enhanced motoneuron survival and regeneration in injured mice treated with L1 Ab 557

**Figure 2:**
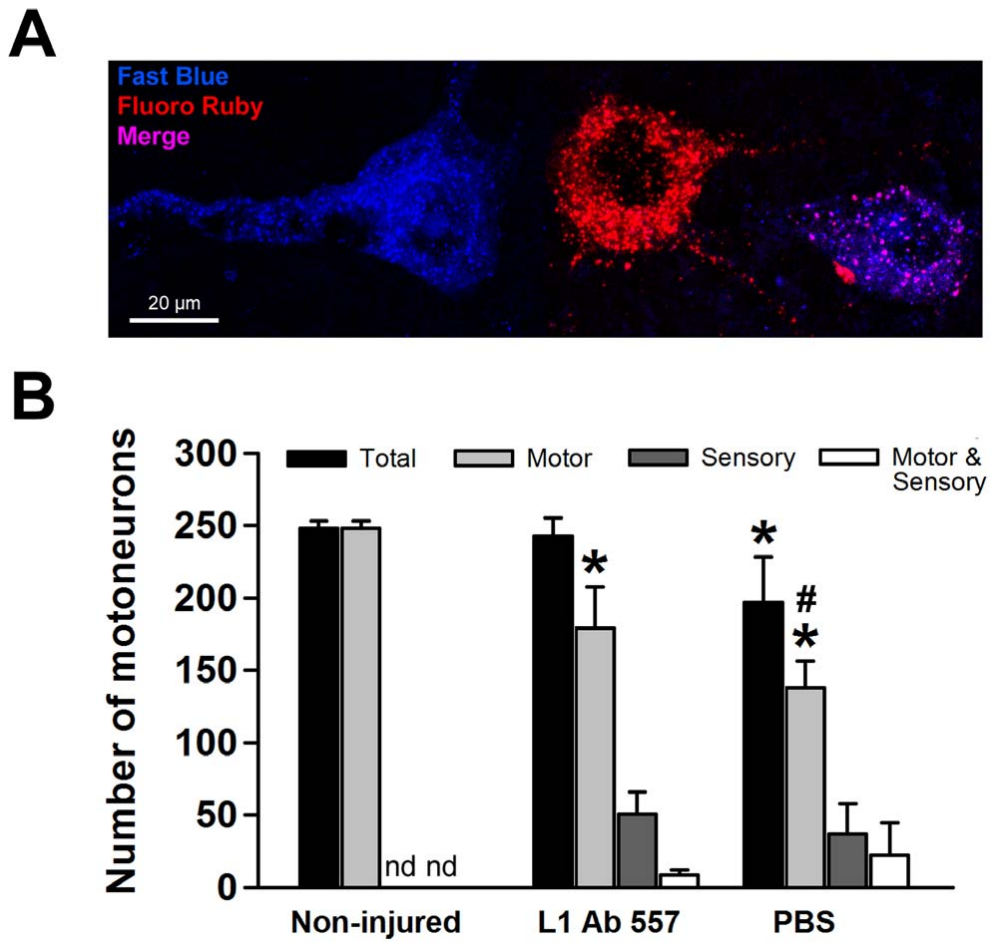
Analysis of axonal regrowth and/or survival of motoneurons. (**A**) Representative image of motoneurons labeled with Fast Blue (blue, correctly regrown into the motor branch), Fluoro Ruby (red, incorrectly regrown into the sensory branch), and merge (violet, regrown into both branches, motor and sensory). (**B**) Mean numbers of regenerated motoneurons (+ SEM) labeled through the motor branch only (light gray bars, “Motor”), the sensory branch only (dark gray bars, “Sensory”) and the sum of neurons labeled both through motor and sensory branches (black bars, “Total”) 12 weeks after injury in mice treated with L1 Ab 557 or PBS. In non-injured mice, retrograde tracing was performed without previous injury. In this group, no incorrectly projecting neurons (white bars, “Motor & Sensory”) are detected (nd). Ten mice per group were analyzed. Asterisks indicate differences in number of motoneurons between non-injured and injured groups. Cross-hatches show differences in number of motoneurons between groups treated with L1 Ab 557 or PBS (*p<0.05, ^#^p<0.05, one-way ANOVA with Tukey's *post hoc* test).

To analyze the numbers of neurons having regrown into the appropriate branch, retrograde labeling of motoneurons was performed at 12 weeks after injury. The total number of motoneurons retrogradely labeled through the motor or the sensory branches, or through both branches of the femoral nerve, was reduced after treatment with PBS compared with non-injured mice and mice treated with L1 Ab 557. The total number of motoneurons in L1 Ab 557 treated mice was similar to that in non-injured mice and displayed preferential motor reinnervation (PMR) when compared with the PBS treated mice (Fig. 2B). In every experimental group, small fractions of all labeled motoneurons had projected inappropriately into the sensory branch only or into both the motor and the sensory branches (Fig. 2A and B). The degree of PMR, estimated as the ratio of motoneurons projecting into the motor branch over motoneurons projecting into the sensory branch, was higher in mice treated with L1 Ab 557 compared to mice having received PBS (13.6±6.6 *versus* 5.4±2.5) (p<0.05, *t* test). Thus, motoneuron loss and/or failure of axonal regrowth are reduced and precision of preferential motor reinnervation is improved in mice treated with L1 Ab 557.

### L1 Ab 557 improves myelination

**Figure 3:**
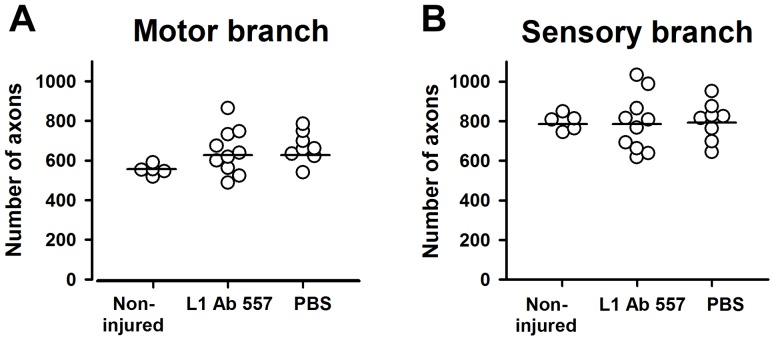
Analysis of myelinated axons in non-injured and regenerated nerve branches. Number of axons in motor (**A**) and sensory (**B**) nerve branches at 12 weeks after injury in mice treated with L1 Ab 557 or PBS and in non-injured contralateral nerves. Ten mice per group were analyzed. No differences are detectable between the experimental groups (p>0.05, one-way ANOVA with Tukey's *post hoc* test).

**Figure 4:**
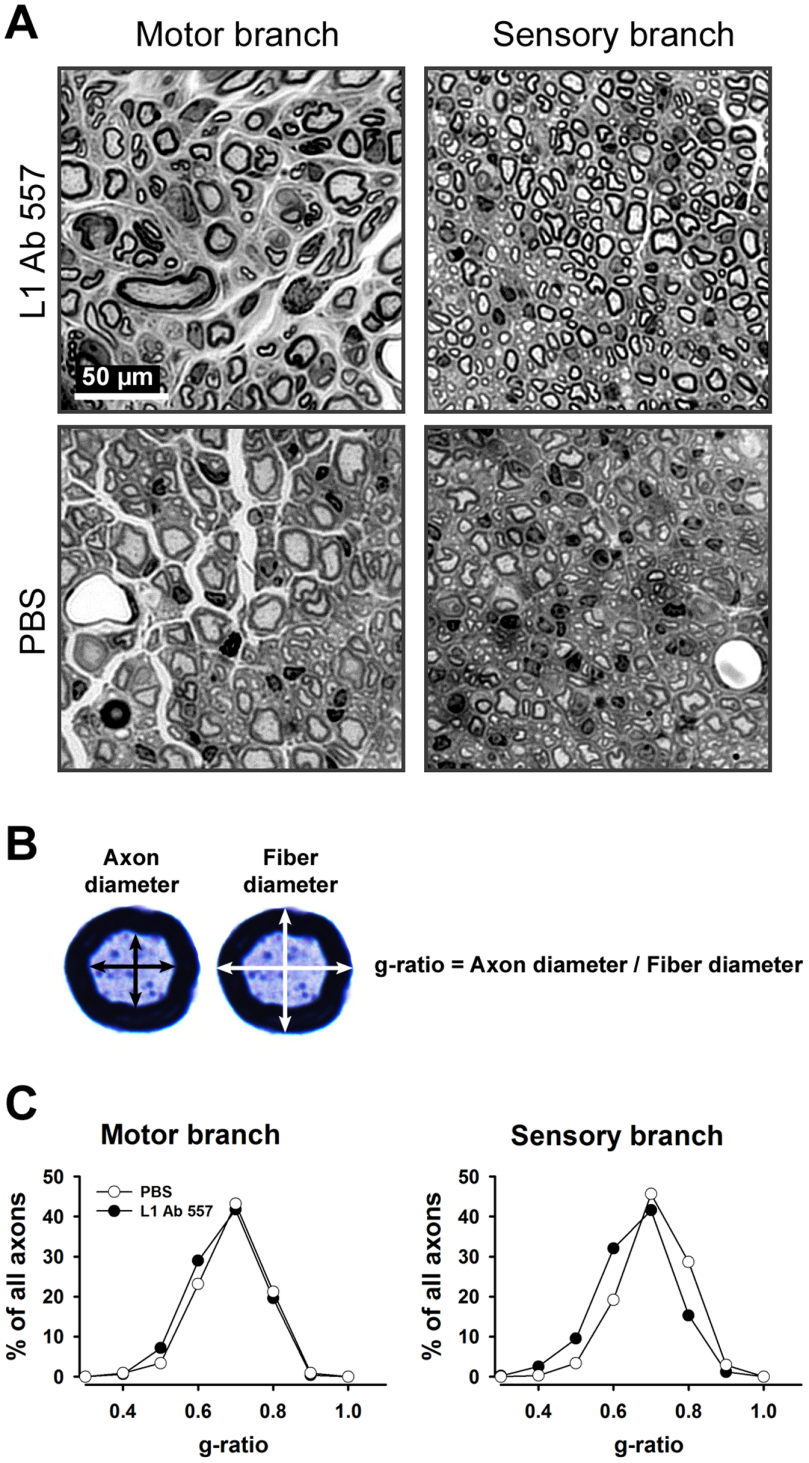
Analysis of myelination in regenerated femoral nerves in mice treated with L1 Ab 557 or PBS in the conduits applied to the transected nerves. (**A**) Representative images of the motor and sensory nerve branches from L1 Ab 557 or PBS treated mice. (**B**) Mean orthogonal diameters of the axon (black arrows) and of the nerve fiber (white arrows) were measured and the degree of myelination was estimated by the ratio of axon to fiber diameter (g-ratio). (**C**) Normalized frequency distributions of g-ratios in regenerated motor and sensory nerve branches. Regenerated nerves were studied 12 weeks after injury. Ten mice per group were analyzed. The shift in distributions of g-ratios to the left in the group treated with L1 Ab 557 in the sensory nerve shows better myelination compared to the group of animals treated with PBS (p<0.05, Kolmogorov-Smirnov test).

**Figure 5:**
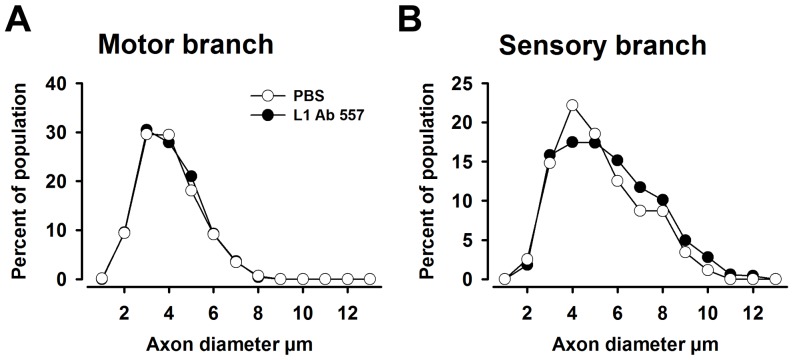
Analysis of axon diameters in regenerated nerves. (**A, B**) Normalized frequency distributions of axon diameters in regenerated motor and sensory nerve branches of the femoral nerve in mice treated with L1 Ab 557 or PBS. Regenerated nerves were studied 12 weeks after injury. (**A**) No difference is seen for the experimental versus control motor branches. (**B**) Differences between groups are detected for the sensory nerve branch. Ten mice per group were analyzed (p<0.05, Kolmogorov-Smirnov test). The shift in distribution of diameters reflects an increased proportion of large-diameter axons in the sensory branch of mice treated with L1 Ab 557.

We next analyzed non-injured and regenerated nerves histologically to assess axonal numbers, axonal diameters and degree of myelination 12 weeks after injury. In both motor and sensory femoral nerve branches, the total number of myelinated axons was similar in both experimental groups compared with non-injured mice (Fig. 3A and B). However, myelin thickness in regenerated nerves was higher in the sensory branches of mice treated with L1 Ab 557 than in mice treated with PBS, as assessed by g-ratio (p<0.05, Kolmogorov-Smirnov test, Fig. 4A–C). Interestingly, the frequency distribution of axonal diameters was shifted towards higher values only in sensory branches in mice treated with L1 Ab 557 (p<0.05, Kolmogorov-Smirnov test) indicating that axons had larger diameters and more myelin compared PBS treated control mice (Fig. 5).

### L1 Ab 557 increases sizes of motoneuron somata and synaptic rearrangements in the spinal cord after femoral nerve injury

**Figure 6:**
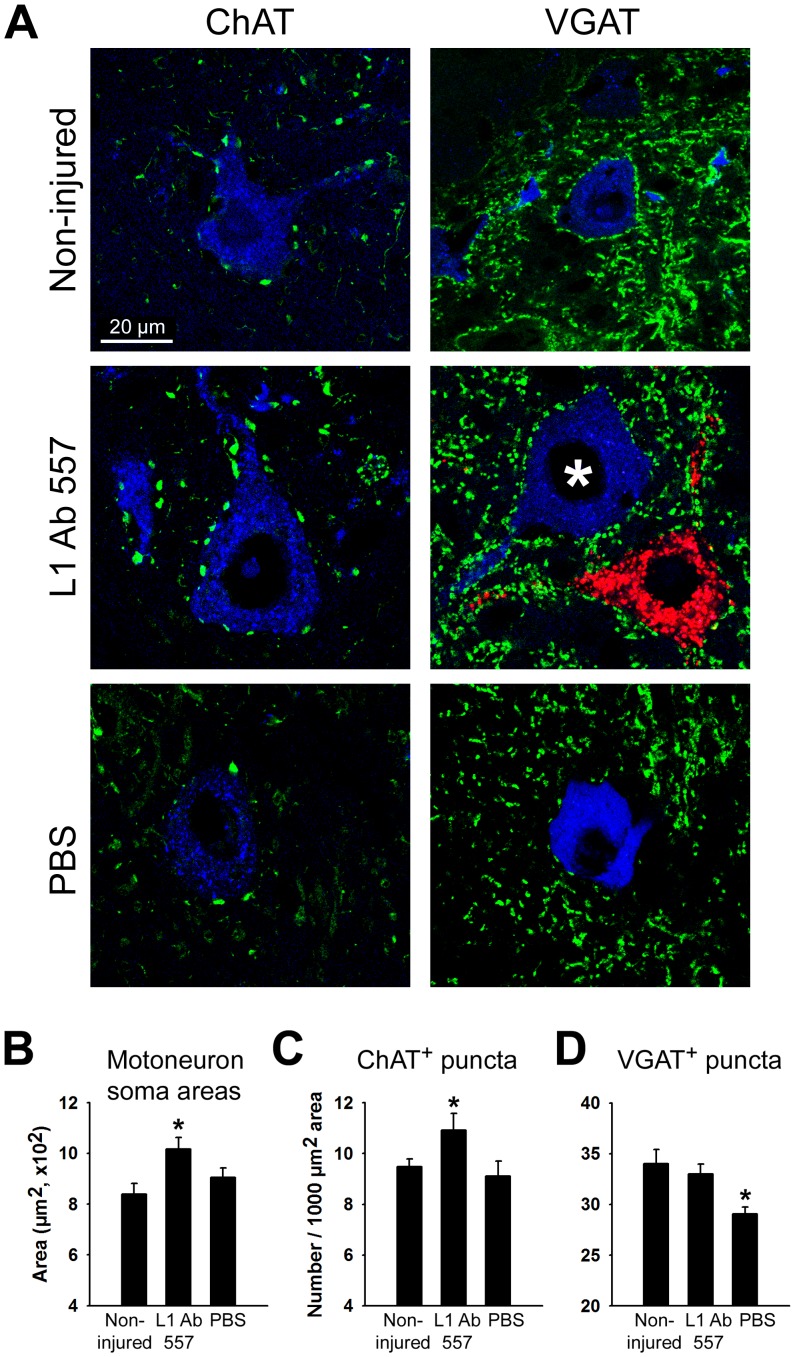
Analysis of motoneuron soma sizes and numbers of perisomatic nerve terminals. (**A**) Representative images of analyzed neurons. Analysis was performed for correctly projecting motoneurons retrogradely labeled with Fast Blue (* – blue soma), but not for neurons labeled with Fluoro-ruby (red soma), after application to the motor and sensory nerve branches, respectively. (**B**) Soma area of ChAT^+^ and VGAT^+^ motoneurons identified by retrograde labeling. (**C, D**) Linear densities of ChAT^+^ and VGAT^+^ puncta in non-injured mice which were subjected to retrograde tracing only and in mice treated with L1 Ab 557 or PBS at 12 weeks after injury. Between 200 and 350 cells from 10 animals were analyzed per group and parameter. Shown are group mean values+SEM calculated from individual mean values (*p<0.05, one-way ANOVA with Tukey's *post hoc* test).

Soma sizes of motoneurons and levels of cholinergic (ChAT^+^) terminals and inhibitory (GABAergic and glycinergic, VGAT^+^) terminals at motoneuron perikarya were measured as indicators for successful recovery at 12 weeks after injury. Somata of motoneurons were larger in L1 Ab 557 treated mice or non-injured mice compared with PBS treated mice (Fig. 6A and B).

Densities of cholinergic (ChAT^+^) terminals at motoneuron perikarya were increased in mice treated with L1 Ab 557 compared with the PBS group or mice without injury (Fig. 6A and C). Densities of GABAergic and glycinergic (VGAT^+^) terminals were decreased in the PBS group as compared with mice treated with L1 Ab 557 and the non-injured group, which were similar (Fig. 6A and D). These findings indicate that structural synaptic plasticity related to nerve repair differs in L1 Ab 557 treated mice *versus* the PBS control group in modulatory cholinergic and inhibitory terminals, with the antibody treated mice showing similar values as the non-injured mice, indicating successful regeneration.

## Discussion

As for the central nervous system of mice, L1 is beneficial in recovery for the peripheral nervous system. Our previous cell culture experiments with soluble beneficial function triggering monoclonal antibody 557 revealed optimal stimulation of neuronal growth and survival at 50 µg/ml or higher concentrations of 557 antibody in the cell culture medium [37], hence 400 µg/ml was chosen for the *in vivo* experiments to assure that the maximal stimulatory effect of the antibody can be reached. In these previous experiments we had used 200 µg/ml of a Fab fragment derived from antibody 557 *in vivo* and shown that the Fab is active in a similar micro-molar range as the IgG. Application of the L1 Ab 557 is favorable for functional recovery after mouse femoral nerve injury as assessed by locomotor and histological analyses: attenuated motoneuron loss as well as enhanced precision of motor reinnervation and myelination were observed with this antibody. Preferential motor reinnervation after injury was more pronounced with L1 Ab 557, thus resembling the phenomenon seen, although slightly less effectively, with mimetics for the HNK-1 glycan, but not with mimetics for alpha 2,8 polysialic acid [33], [38]. Our observations *in vivo* are in agreement with those on the *in vitro* application of L1 Ab 557 or an Fab fragment derived from L1 Ab 557, leading to enhanced neurite outgrowth, but differ from observations with polysialic acid mimetics, which demonstrate enhanced neurite outgrowth, but no preferential motor reinnervation [38], [44], [45]. These results suggest that stimulation of neurite outgrowth and precision of reinnveration most likely occur via independent mechanisms, which are triggered by L1 Ab 557 and HNK-1 mimetics, but not by PSA or PSA mimetics, which merely stimulate neurite outgrowth, but do not lead to preferential motor reinnervation. Thus, increased regrowth of axons cannot be the reason for increased preferential motor reinnervation triggered by L1 Ab 557.

Enhanced axonal regrowth is paralleled by enhanced soma size of regenerated motoneurons, indicating positive effects on motoneuron survival after axotomy, a phenomenon that has been related to increased production of growth and neuroprotective factors [46]. The neuroprotective function known for L1 [47] is likely to contribute also to enhanced motoneuron survival and enhanced soma size of motoneurons, probably also due to enhanced production of neurotrophic factors and stimulation of anti-apoptotic pathways.

With reinnervation, motoneuron cell bodies recover synaptic input [48]. Indeed, L1 Ab 557 changes the numbers of inhibitory (GABAergic and glycinergic, VGAT^+^) terminals and modulatory cholinergic (ChAT^+^) terminals at motoneuron perikarya. Cholinergic synaptic input to motoneuron perikarya modulates the excitability of motoneurons and plays an important role in the control of locomotion [49]. Increased numbers of cholinergic (ChAT^+^) boutons correlate with maintenance of excitatory synapses which contribute to improved recovery after injury [50]. Our findings are in agreement with the findings on L1's capacity to stimulate ChAT activity *in vitro* [51], at least partially regulated through the fibroblast growth factor receptor [2], which is known to activate the expression of ChAT [52]. Inhibitory input via GABA and glycine receptors balances skeletal muscle activity [53], [54]. In the present study, L1 Ab 557 increased numbers of perisomatic, spinal interneuron-derived inhibitory (VGAT^+^) terminals, similar in extent to the non-injured situation. Interestingly, L1 activation is necessary for its interaction with the cytoskeletal linker molecule ankyrin [55]–[58] that regulates neurite extension and branching, and stabilizes perisomatic synapses of GABAergic interneurons [59], thereby stimulating perisomatic inhibition of axo-somatic synapses on interneurons and motoneurons which is crucial for normal motoneuron function [60].

Our findings also agree with observations in a central nervous system injury paradigm, where treatment of the severed spinal cord with Fab fragments from L1 Ab 557 enhanced regeneration [45]. Furthermore, application of polyclonal L1 antibody and a homophilically acting recombinant L1 fragment as well as ectopic induction of L1 expression in astrocytes increased the number of axons crossing into a monolayer of astrocytes, which are considered a poor substrate for neuritogenesis [61]. *In vivo*, L1 overexpressing embryonic stem cells, neural stem cells or radial glial cells not only enhanced locomotor recovery after spinal cord injury, but also rescued imperiled host motoneurons and parvalbumin-positive interneurons [11]. Furthermore, increased numbers of catecholaminergic nerve fibers distal to the lesion site were seen as well as increased soma size, cholinergic synaptic coverage of host motoneurons and numbers of endogenous catecholaminergic nerve fibers caudal to the lesion site [62], [63]. We interpret our findings in that L1 antibody application to the injured femoral nerve may attenuate Schwann cell differentiation into the myelinating phenotype and enhance Schwann cell process formation and elongation, thereby favoring the secretion of neurotrophic factors by non-differentiated Schwann cells and enhancing remyelination. We suggest that L1 overexpression in the peripheral nervous system may not only lead to stimulation of L1-mediated signaling pathways, but also to interaction with heterophilic partners which promote beneficial functions. It is noteworthy in this respect that L1 is most prominently expressed by axons during axonal outgrowth, and less by dendrites and cell bodies [64]. After injury to the adult central nervous system, L1 expression is upregulated in neurons capable of regeneration, such as retinal ganglion cells [65], [66] and neurons in the thalamic reticular nucleus [67], substantia nigra [68], brain stem and cerebellar deep nuclei [69]. In contrast, Purkinje cell neurons, corticospinal tract neurons and striatal neurons with low intrinsic regenerative capability fail to upregulate L1 expression after axotomy [67]–[69].

In the PNS, L1 is detected on both non-myelinating Schwann cells and non-myelinated sensory axons of adult mice. Non-myelinating Schwann cells show a reduced association with axons in L1 knock-out mice, and the non-myelinated sensory axons undergo degeneration [18], [26], [70], [71], suggesting that axonal L1 regulates Schwann cell-axon adhesion of non-myelinated fibers. Homophilic L1 interactions promote outgrowth of neurons on cultured Schwann cells and enhance Schwann cell motility [29], [71]–[73]. Furthermore, results from sciatic nerve transplantation experiments suggest that axonal L1 is responsible for maintenance of sensory axon-Schwann cell ensheathment by a heterophilic adhesion mechanism between axonal L1 and a Schwann cell molecule [26]. Experiments using L1 knock-in mice, in which the 6^th^ immunoglobulin domain is deleted, indicate that interactions of L1 on axons with integrins on Schwann cells may be essential for ensheathment and may affect myelination [74]. Enhancement in neurite outgrowth is not only achieved with substrate-coated extracellular matrix molecules or extracellular domain of L1, but also with antibodies against fibronectin type III like domains of L1 [35], [45]. Interestingly, antibodies against the immunoglobulin-like domains of L1 have the opposite effect: they inhibited Schwann cell ensheathment of neurons *in vitro* [22], [75]. These results show that, depending on the binding site of L1 antibodies, L1 mediated functions can be triggered or inhibited. Similar observations were reported for antibodies against the neuronal cell surface molecule Thy-1 [76] and for a monoclonal antibody that recognizes the beta subunit of integrin [77]. Moreover, in a Schwann cell-astrocyte-boundary culture model, application of an L1 antibody and L1-Fc, or adenoviral transduction of L1 into astrocytes increased the proportion of dorsal root ganglia axons able to overcome the astrocytic boundary [61]. These results indicate that heterophilic and homophilic L1 interactions between neurons and Schwann cells play an important role during myelination.

In the present study we observed better myelination in the sensory branch of the femoral nerve of mice treated with L1 Ab 557, while the myelination in the motor branch did not differ from the PBS treated group. This finding might be related to a more pronounced expression of L1 by sensory but not motor axons [26] and could explain the more pronounced L1-mediated myelination in the sensory branch. Our findings could also explain the larger axonal diameters in the sensory, but not motor branch after the local application of L1 Ab 557.

The mechanisms by which L1 Ab 557 triggers neurite outgrowth are noteworthy in that the epitope recognized by this antibody recruits second messenger systems in neurons and Schwann cells, such as Ca^2+^ or inositol-1,4,5-trisphosphate [35]. In a similar *in vitro* paradigm [78], L1-mediated neurite outgrowth was completely inhibited by Ca^2+^-channel blockers and pertussis toxin. Partial inhibition of neurite outgrowth in these experiments suggests that different molecular mechanisms are involved [35], [79]. These may comprise Ca^2+^ and G-protein independent second messenger cascades or even modes of signal transduction independent of second messenger systems. For instance, application of L1 Ab 557 leads to sumoylation and proteolytic processing of L1 [36] which is required for L1-mediated neuritogenesis and neuronal survival *in vitro* [80], suggesting that proteolysis of L1 and activation of second messenger cascades in conjunction with gene transcription underlie better functional recovery *in vivo*.

Studies related to regeneration in the presence of L1 antibody have to be taken with caution with regard to systemic application, since L1 is not only expressed in the nervous system but also, for instance, by cells of the immune system, skin, kidney and intestine and application of function triggering L1 antibodies could lead to stimulation of L1-dependent functions in these cells, thereby inducing side effects, which could be avoided by local antibody application. Our study with direct application of beneficial function-triggering L1 antibody indicates favorable effects on recovery and may encourage designing novel, clinically feasible strategies to improve functional outcomes after nervous system injury, for instance by construction of biodegradable conduits and particles that allow controlled release of L1 function triggering reagents, not only the peripheral but also central nervous system.
